# Clinical efficacy of icotinib in lung cancer patients with different *EGFR* mutation status: a meta-analysis

**DOI:** 10.18632/oncotarget.15475

**Published:** 2017-02-18

**Authors:** Jian Qu, Ya-Nan Wang, Ping Xu, Da-Xiong Xiang, Rui Yang, Wei Wei, Qiang Qu

**Affiliations:** ^1^ Department of Pharmacy, the Second Xiangya Hospital, Central South University, Institute of Clinical Pharmacy, Central South University, Changsha 410078, P.R.China; ^2^ Department of Respiratory, Hospital of Laiwu Iron and Steel Co.Ltd, Laiwu 271100, P.R.China; ^3^ Department of Pharmacy, Xiangya Hospital, Central South University, Changsha 410078, P.R.China; ^4^ Department of General Surgery, Xiangya Hospital, Central South University, Changsha 410008, P.R.China

**Keywords:** icotinib, epidermal growth factor receptor, meta-analysis, objective response rate, disease control rate

## Abstract

Icotinib is a novel and the third listed epidermal growth factor receptor-tyrosine kinase inhibitors (EGFR-TKIs), which exerts a good anti-tumor efficacy on non-small cell lung cancer (NSCLC). The efficacy of EGFR-TKIs has been shown to be associated with the *EGFR* mutation status, especially exon 19 deletion (19Del) and exon 21 L858R mutation. Therefore, a meta-analysis was performed to assess the efficacy of icotinib in NSCLC patients harboring *EGFR* mutations (19Del or L858R) and wild type (19Del and L858R loci wild type). A total of 24 studies were included for comparing the objective response rate (ORR) in the *EGFR* wild type and mutant patients treated with icotinib. The ORRs of *EGFR* mutant patients (19Del or L858R) are better than those of *EGFR* wild type patients (OR = 7.03(5.09–9.71), *P* < 0.00001). The pooling ORs from 21 studies on the disease control rate (DCR) in *EGFR* mutant patients are better than those of *EGFR* wild type patients (OR = 10.54(5.72–19.43), *P* < 0.00001). Moreover, the ORRs of *EGFR* 19Del patients are better than those of *EGFR* L858R patients after pooling ORs of 12 studies (OR = 2.04(1.12–3.73), *P* = 0.019). However, there was no significant difference on DCRs of *EGFR* 19Del patients and those of *EGFR* L858R patients (OR = 2.01(0.94–4.32), *P* = 0.072). Our findings indicated that compared with *EGFR* wild type patients, *EGFR* mutant patients have better ORRs and DCRs after icotinib treatment; *EGFR* 19Del patients treated with icotinib have better ORRs than *EGFR* L858R patients. *EGFR* mutation status is a useful biomarker for the evaluation of icotinib efficacy in NSCLC patients.

## INTRODUCTION

Lung cancer is the leading cause of mortality around the world and non-small cell lung cancer (NSCLC) is up to 85% of all types of lung cancer [1]. NSCLC mainly includes squamous cell carcinoma, adenocarcinoma and large cell carcinoma. No obvious clinical manifestations were observed at early stage and more than 40% of NSCLC are metastatic (Stage IV) disease at diagnosis [2].

With in-depth studies of genesis and cancer related signal pathway, epidermal growth factor receptor (EGFR)-dependent pathway was revealed to play important roles in the development and progression of epithelial cells in NSCLC patients [3]. EGFR tyrosine kinase inhibitors (EGFR-TKIs) play important roles in the treatment of advanced NSCLC because of their superior efficacy over than chemotherapy [4]. EGFR-TKIs such as gefitinib and erlotinib were identified to extend survival and increase quality of life in NSCLC patients [4–6]. Icotinib was a novel and the third listed EGFR-TKIs, which could exert a good anti-tumor efficacy on NSCLC [7],especially in the re-treatment of advanced NSCLC [8]. Up to now, it has become one of the standard drugs for the treatment of advanced NSCLC in China [9].

The efficacy of EGFR-TKIs has been shown to be associated with the *EGFR* mutation status, especially exon 19 and exon 21 that are sensitive to targeted drug therapies [4, 7, 10–15]. Moreover, previous studies has revealed that patients treated with icotinib harboring *EGFR* exon 19 deletion (19Del) had better survival than those harboring exon 21 point mutation (L858R) [16] or there were no difference among the patients harboring 19Del or L858R mutations [7, 17, 18]. Because of the inconsistent results, relative small sample sizes and lack of high quality studies, their conclusions are limited value. Therefore, we reviewed all the publications about icotinib and conducted a meta-analysis to assess the efficacy of icotinib in NSCLC patients harboring *EGFR* mutations (19Del or L858R) or wild type for these two mutations

## RESULTS

### Study review and selection

**Figure 1:**
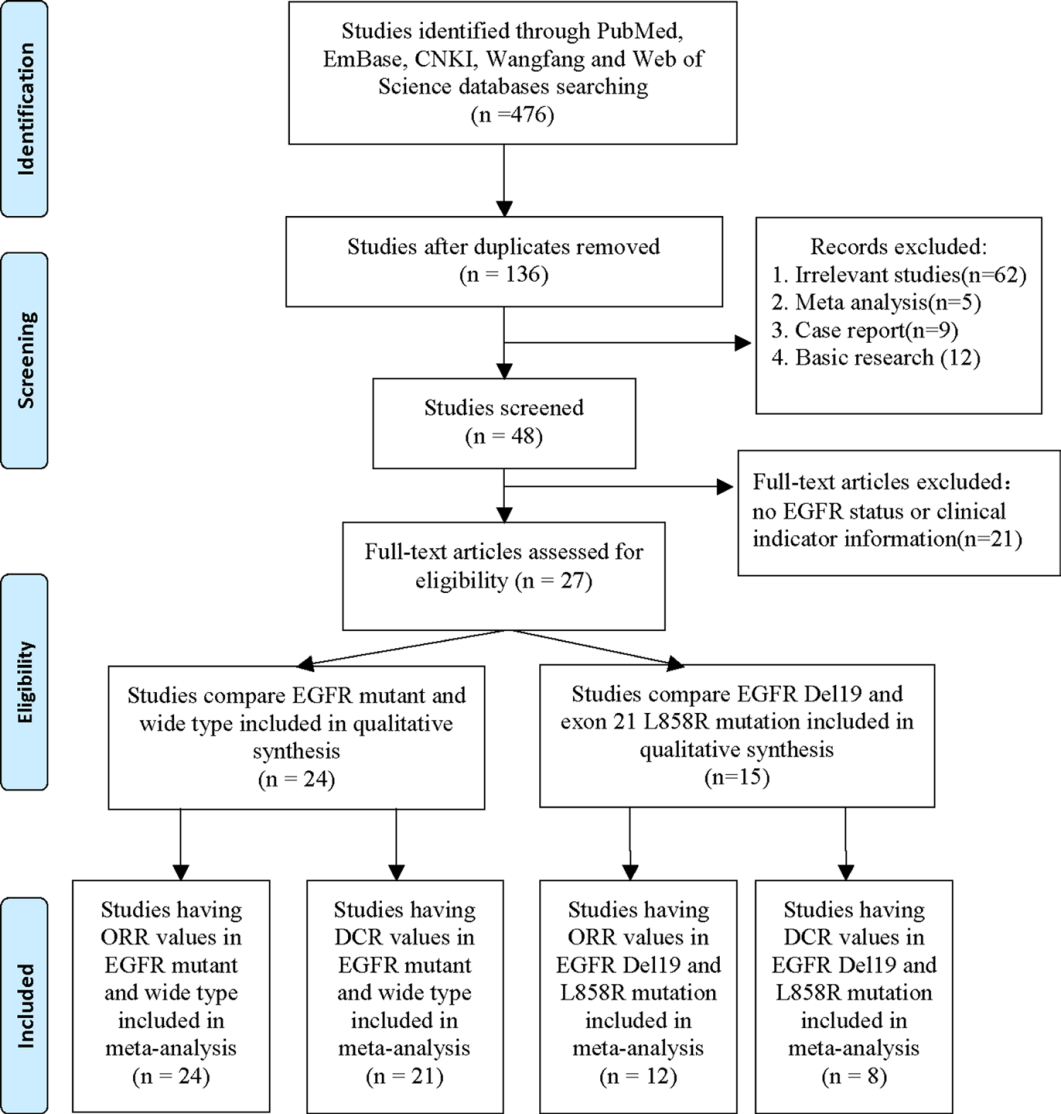
Procedure of article selection

The study selection procedure is shown in Figure 1. We searched from the databases including PubMed, EMBASE, Web of Science, Wanfang and Chinese National Knowledge Infrastructure (CNKI) to 14th Oct. 2016. A total of 136 publications were found after excluding the duplicated studies. Then we excluded 62 irrelevant studies, 5 meta-analysis, 9 case reports and 12 basic studies. Forty-eight studies were included for further review. We further excluded the article that has no *EGFR* status or no clinical indicators including objective response rate (ORR) and disease control rate (DCR). Twenty-four publications having *EGFR* mutation and wide type data were selected to qualitative synthesis. Fifteen studies having *EGFR* 19Del and L858R mutation data were included in qualitative synthesis. Finally, 24 studies having ORR values and 21 studies having DCR values in *EGFR* mutant and wild type patients were enrolled for meta-analysis. Twelve studies having ORR values and 8 studies having DCR values in *EGFR* 19Del and L858R patients were enrolled for meta-analysis.

### Characteristics of included studies

Not all studies had all clinical indicators data. After summarized, there were 24 publications have ORR data compared with *EGFR* mutation and wild type patients. Among the 24 publications, except one publication [19], 23 publications have DCR data. There were 12 publications having ORR values and 8 publications having DCR values in *EGFR* 19Del and L858R patients (Table 1). The characteristics of first author's name, publishing year, region, Newcastle-Ottawa Scale (NOS) score, study design and the number of patients harboring *EGFR* mutation status were shown in Table 1.

**Table 1:** Characteristics of studies included in meta-analysis

Author	Year	Region	Study design	NOS Score	*EGFR* mutant and wild type			*EGFR* 19Del and L858R			References
					M/W number	ORR	DCR	19Del/L858 number	ORR	DCR	
Zhao Q	2011	China	R	6	5/14	Y	Y	4/1	Y	N	[20]
Zen Xiao-Mei	2013	China	R	6	15/3	Y	Y		N	N	[38]
Yang Xin-Jie	2013	China	R	6	18/2	Y	Y	10/8	Y	Y	[39]
Yang S	2016	China	R	7	32/55	Y	N		N	N	[31]
Wei Feng-Lu	2015	China	R	6	15/15	Y	Y		N	N	[40]
Wang Tao	2016	China	R	7	38/29	Y	Y	15/16	Y	N	[32]
Tan Fen-Lai	2012	China	R	7	249/101	Y	Y	72/88	Y	Y	[35]
Sun Jing	2014	China	R	7	34/10	Y	Y		N	N	[41]
Song Zheng-Bo	2013	China	R	6	36/13	Y	Y		N	N	[42]
Shao L	2014	China	R	7	12/16	Y	Y		N	N	[43]
Ren G-J	2011	China	R	5	7/7	Y	Y	3/4	Y	Y	[44]
Pang Lin-Rong	2014	China	R	6	33/18	Y	Y	19/13	Y	Y	[21]
Nong Jin-Yin	2013	China	R	6	23/9	Y	Y	14/9	Y	N	[22]
Na Qin	2013	China	R	6	35/11	Y	Y		N	N	[16]
Ma Xiang-Leiei	2014	China	R	7	40/14	Y	Y		N	N	[45]
Liang Shao-Ping-Ping	2015	China	R	5	10/2	Y	Y		N	N	[46]
Li Xi	2012	China	R	6	23/36	Y	Y	55/40	Y	Y	[34]
Li Xi	2015	China	R	6	99/25	Y	Y	13/9	Y	Y	[18]
Li Ran	2013	China	R	5	23/7	Y	Y	17/6	Y	Y	[47]
He Xiao-Tin	2015	China	R	6	33/13	Y	Y		N	N	[48]
He Chun-Xiao	2012	China	R	7	1/4	Y	Y		N	N	[49]
Guo Lei	2016	China	R	7	21/6	Y	Y		N	N	[50]
Gu A	2013	China	R	5	4/2	Y	Y		N	N	[51]
Chen Xiao-Feng	2014	China	R	6	19/63	Y	Y		N	N	[33]
Zhang Xiao-Xue	2016	China	R	7		N	N	60/44	Y	N	[7]
Shen Yan-Wei	2016	China	R	6		N	N	21/14	Y	Y	[17]

Abbreviations: Y, Yes; N, NO; M, Mutation; W, wild type; Del, deletion; R, Retrospective study; NOS, Newcastle-Ottawa Scale; ORR, Objective Response Rate; DCR, disease control rate.

### Quality evaluation of enrolled publications

**Figure 2:**
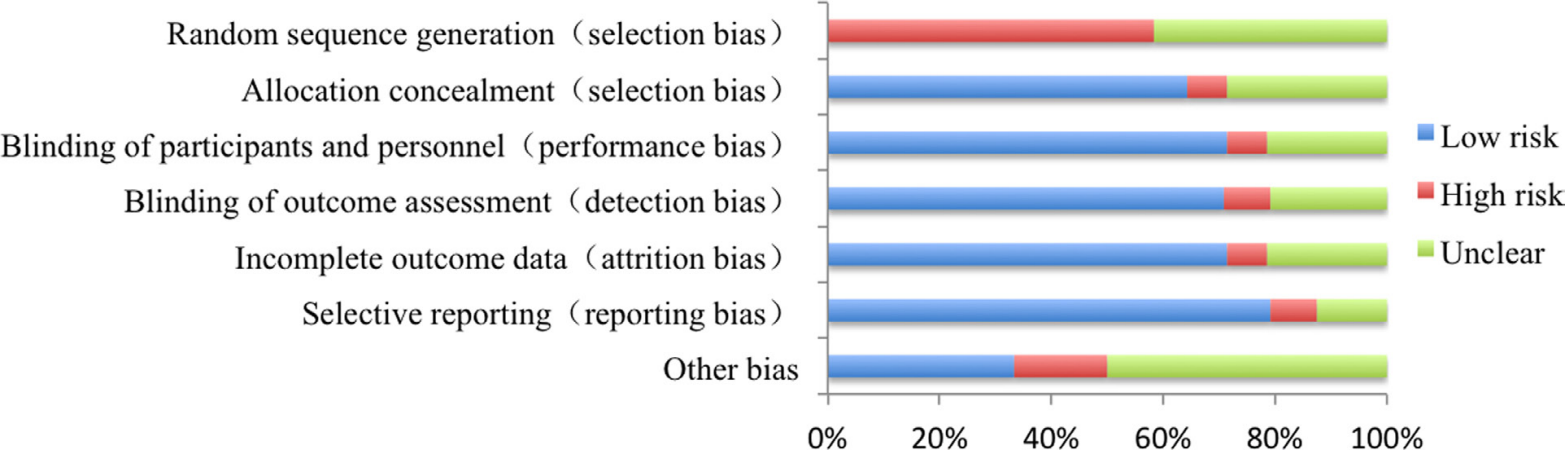
Analysis of risk of bias

The details of the quality evaluation of enrolled publications were shown in Figure 2. There were no randomized controlled trials involved in *EGFR* status on the efficacy of icotinib in NSCLC patients harboring different *EGFR* mutation status. All studies were cohort trials and retrospective studies. Therefore the risk of bias was high regarding adequate sequence generation and blinding. But other methodological issues present relatively little risk.

### *EGFR* wild type *vs*. *EGFR* mutation

**Figure 3:**
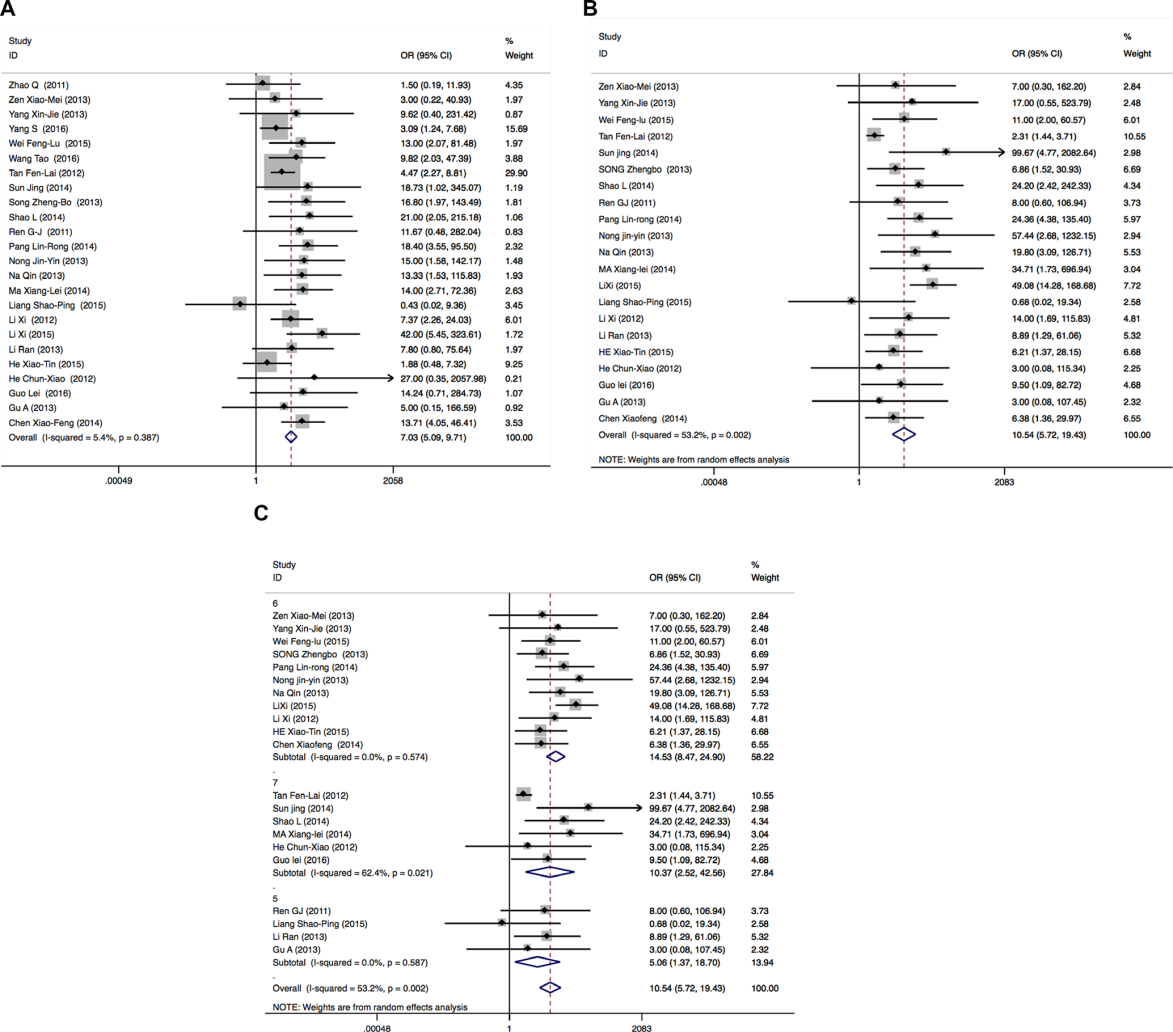
Forest plots of studies evaluating odds ratios of ORRs (**A**), DCRs (**B**) and DCRs subgroups analysis according to NOS (**C**) in *EGFR* wild type and *EGFR* mutant patients. OR: odds ratio.

There were 24 publications enrolled for comparing the ORR in *EGFR* wild type patients and *EGFR* mutant patients (19Del or L858R). Total patients enrolled in the meta-analysis were 1300 including 825 *EGFR* mutant patients and 475 *EGFR* wild type patients. There was no heterogeneity among 24 publications (I^2^ = 5.4%, *P* = 0.387). Therefore, Mantel-Haenszel fixed effects model was used to calculate the pooled odds ratio of included studies. The results showed that the ORRs of *EGFR* mutant patients are better than those of *EGFR* wild type patients (OR = 7.03 (5.09–9.71), *P* < 0.00001) (Figure 3A).

Among the 24 publications, one publication [19] has no data for DCRs; DCRs were 100% in *EGFR* mutant patients and *EGFR* wild type patients in two publications [12, 20]. After excluding these 3 publications, 21 publications having 1127 patients including 750 *EGFR* mutant patients and 377 *EGFR* wild type patients were enrolled in meta-analysis for DCRs. Because of heterogeneity (I^2^ = 53.2%, *P* = 0.002), Mantel-Haenszel random effects model was used to analyze. The results showed that the DCRs of *EGFR* mutant patients are better than those of *EGFR* wild type patients (OR = 10.54 (5.72–19.43), *P* < 0.00001)(Figure 3B). According to subgroup analysis for NOS, there was no heterogeneity in NOS 5 and 6 groups (I^2^ = 0.0%, *P* = 0.587; I^2^ = 0.0%, *P* = 0.574, respectively), but NOS 7 groups has heterogeneity (I^2^ = 62.4%, *P* = 0.021). The DCRs of *EGFR* mutant patients are better than those of *EGFR* wild type patients according to subgroups for different NOS (OR = 5.06(1.37–18.7), *P* = 0.015; 14.53(8.47–24.9), *P* < 0.0001; 10.37(2.25–42.56), *P* = 0.001, respectively) (Figure 3C).

### *EGFR* 19Del *vs*. *EGFR* L858R

**Figure 4:**
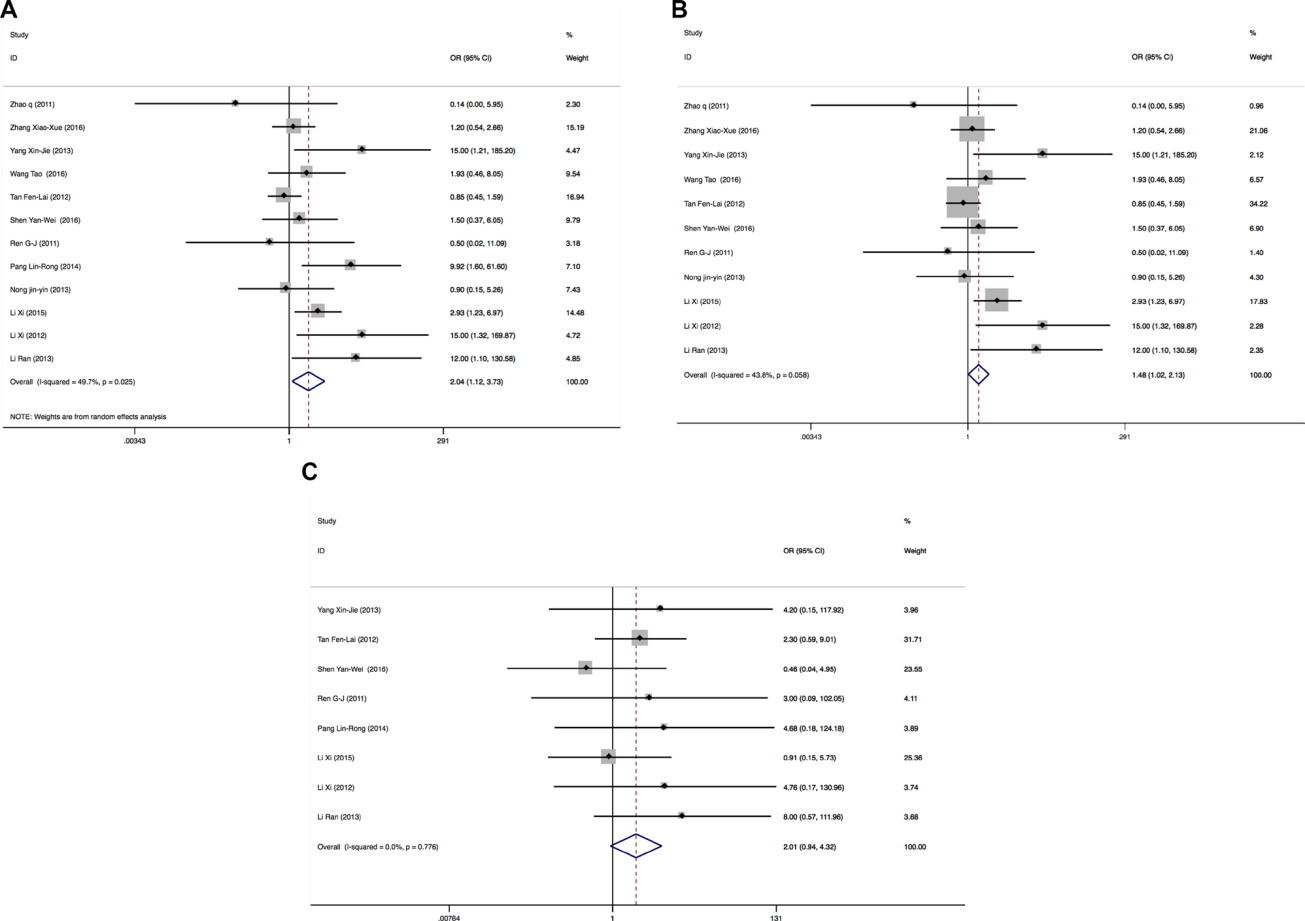
Forest plots of studies evaluating odds ratios of ORRs (**A**), forest plots of studies excluding one publication that influences the overall effective size evaluating odds ratios of ORRs (**B**) and DCRs (**C**) in EGFR 19Del and L858R patients. OR: odds ratio.

There were 12 publications enrolled for comparing the ORR in *EGFR* 19Del and *EGFR* L858R patients. Total patients enrolled in the meta-analysis on ORRs were 555, including 303 *EGFR* 19Del patients and 252 L858R patients. Because of heterogeneity (I^2^ = 49.7%, *P* = 0.025), a Mantel-Haenszel random-effects model was used to analyze. The results showed that the ORRs of *EGFR* 19Del patients are better than those of *EGFR* L858R patients (OR = 2.04 (1.12–3.73), *P* = 0.019) (Figure 4A). After excluded one publication [21] that could influence the overall effective size, there was no heterogeneity (I^2^ = 43.8%, *P* = 0.058) and the ORRs of *EGFR* 19Del patients are better than those of *EGFR* L858R patients (OR = 1.48 (1.02–2.13), *P* = 0.037)) (Figure 4B).

Among the 12 publications, one publication [7] has no data for DCRs; DCRs were 100% in *EGFR* 19Del and L858R patients in three publications [12, 20, 22]. After excluded these 4 publications, 8 publications containing 392 patients including 210 *EGFR* 19Del patients and 182 L858R patients were enrolled in meta-analysis for DCRs. Because there was no heterogeneity (I^2^ = 0.0%, *P* = 0.776), a Mantel-Haenszel fixed-effects model was used to analyze. The results showed that there were no significant differences on DCRs of *EGFR* 19Del patients and L858R patients (OR = 2.01 (0.94–4.32), *P* = 0.072) (Figure 4C).

### Publication bias and sensitivity analysis

**Figure 5:**
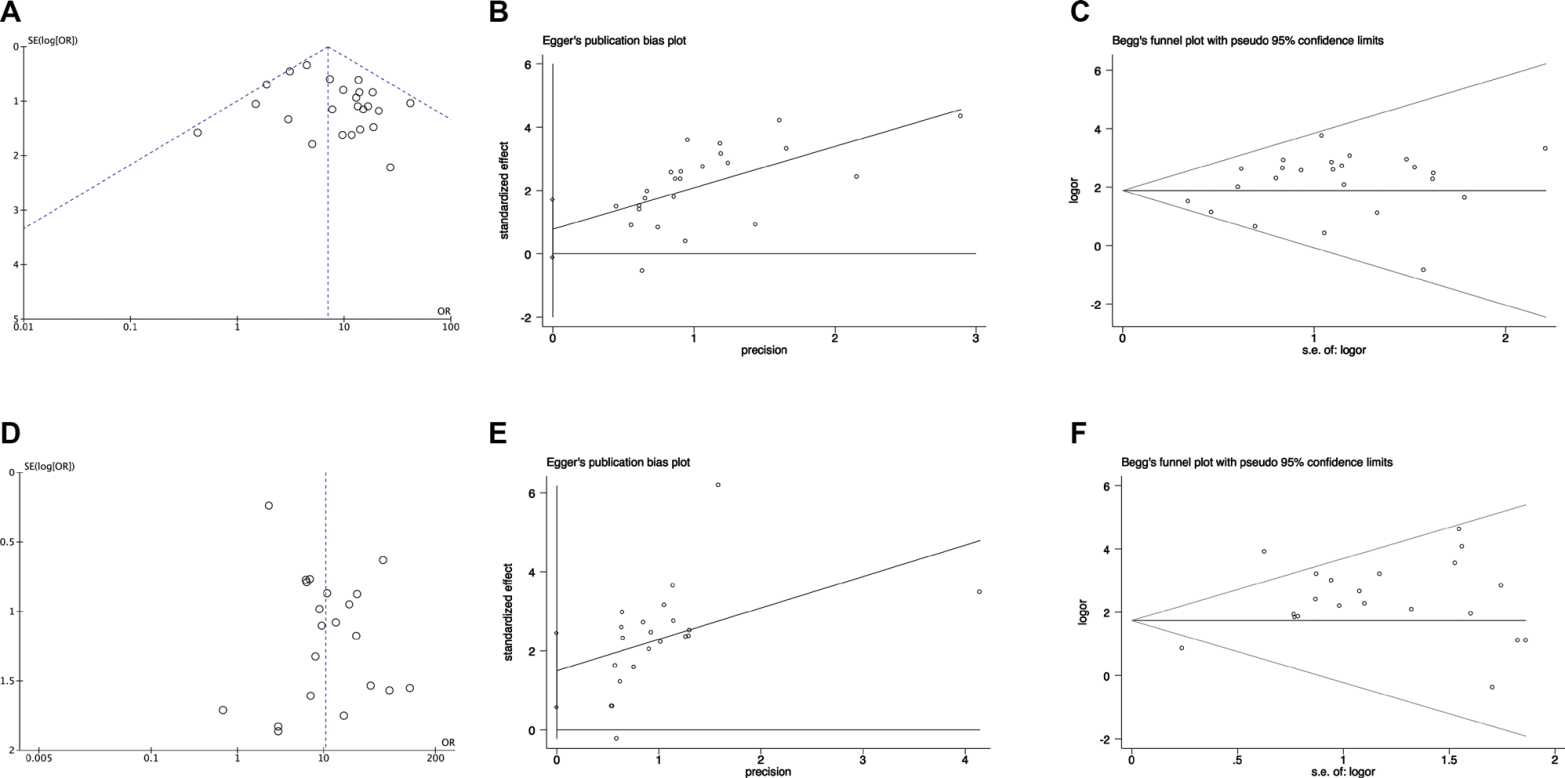
The funnel plots, Egger's test and Begg's test of publication bias in pooling ORs analysis in *EGFR* wild type and *EGFR* mutant patients (**A**) The funnel plots, (**B**) Egger's test and (**C**) Begg's test for pooling ORs of ORRs analysis; (**D**) The funnel plots, (**E**) Egger's test and (**F**) Begg's test for pooling ORs of DCRs analysis. OR: odds ratio; SE: standard error.

Publication bias was examined by funnel plots, Egger's test and Begg's test. For pooling ORs analysis for ORRs in *EGFR* wild type patients and mutant patients, funnel plots, Egger's test (*P* = 0.09) and Begg's test (*P* = 0.747) showed no publication bias (Figure 5A–5C respectively). For pooling ORs analysis for DCRs in *EGFR* wild type patients and mutant patients, funnel plots and Begg's (*P* = 0.651) test showed no publication bias (Figure 5D and 5F, respectively). However, Egger's test (*P* = 0.003) showed publication bias (Figure 5E).

**Figure 6:**
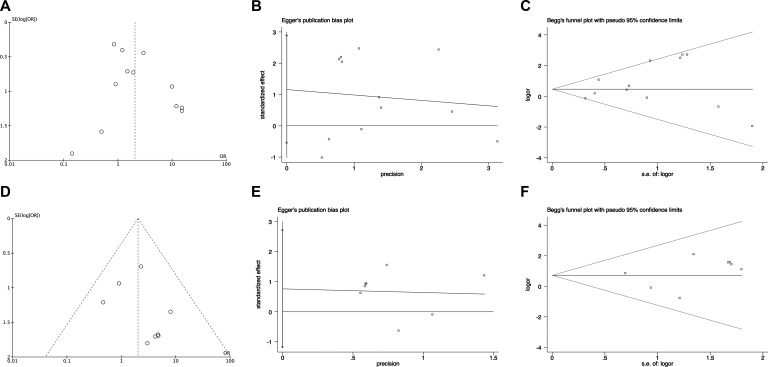
The funnel plots, Egger's test and Begg's test of publication bias in pooling ORs analysis in *EGFR* 19Del and L858R patients (**A**) The funnel plots, (**B**) Egger's test and (**C**) Begg's test for pooling ORs of ORRs analysis; (**D**) The funnel plots, (**E**) Egger's test and (**F**) Begg's test for pooling ORs of DCRs analysis. OR: odds ratio; SE: standard error.

For pooling ORs analysis for ORRs among *EGFR* 19Del and L858R patients, funnel plots, Egger's test (*P* = 0.164) and Begg's test (*P* = 0.451) showed no publication bias (Figure 6A–6C, respectively). For pooling ORs analysis for DCRs in the *EGFR* 19Del and L858R patients, funnel plots, Egger's test (*P* = 0.376) and Begg's test (*P* = 0.711) showed no publication bias (Figure 6D–6F, respectively).

**Figure 7:**
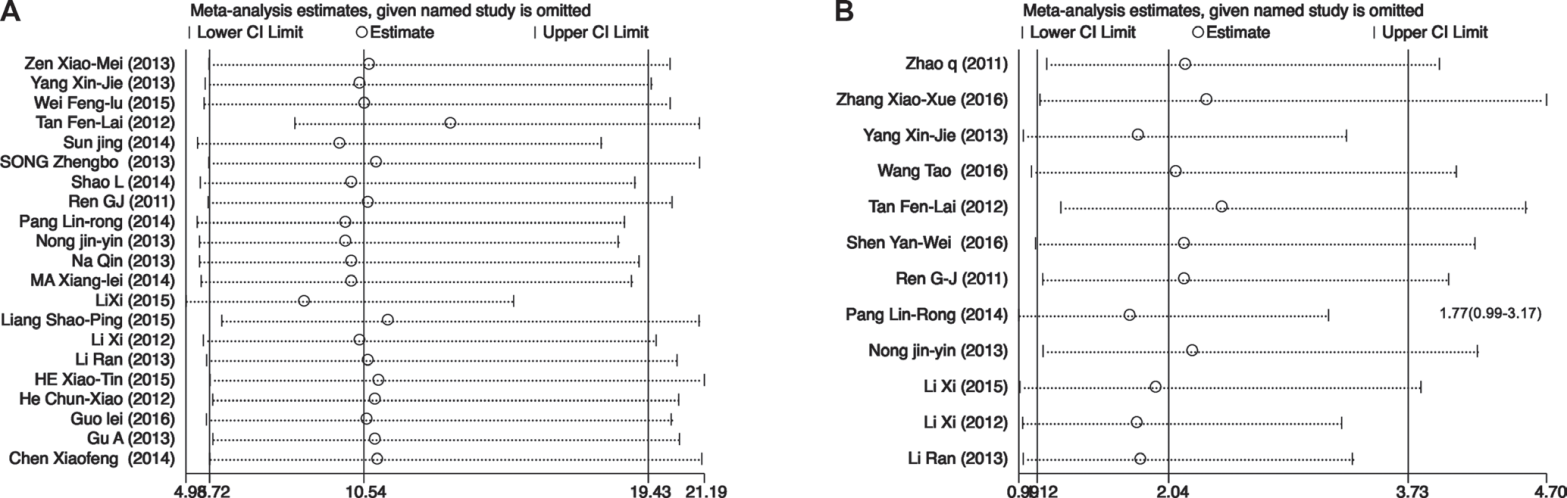
The sensitivity analysis of pooling ORs of DCRs in *EGFR* mutant patients vs. *EGFR* wild type patients (**A**) and ORRs in EGFR 19Del and EGFR L858R patients (**B**). CI: confidence interval.

Sensitivity analysis results showed that changing the effect models had no significant effects on the pooled ORs and the final strength of the association between *EGFR* polymorphisms and clinical efficacy of icotinib in lung cancer patients. Moreover, Figure 7A showed the results of sensitivity analysis regarding DCRs of *EGFR* mutant patients *vs*. *EGFR* wild type patients. We found that excluded studies did not influence the overall effective size in DCRs analysis of *EGFR* mutant patients *vs*. *EGFR* wild type patients. Figure 7B showed one publication (Pang Lin-Rong, 2014) [21] could influence the overall effective size in ORRs analysis of *EGFR* 19Del and *EGFR* L858R patients. After we excluded this publication [21], There was no heterogeneity for pooling ORs analysis for ORRs in *EGFR* 19Del and *EGFR* L858R patients (I^2^ = 43.8%, *P* = 0.058) (Figure 3B).

## DISCUSSION

We carried out this meta-analysis to evaluate the clinical efficacy of icotinib in lung cancer patients with different *EGFR* mutation status. Our meta-analysis suggests that compared with *EGFR* wild type patients, *EGFR* mutant patients have better ORRs and DCRs after icotinib treatment; compared with *EGFR* L858R patients, *EGFR* 19Del patients have better ORRs after icotinib treatment.

It is widely accepted that *EGFR* plays important roles in tumor cell proliferation, invasion, angiogenesis, metastasis and apoptosis [23]. EGFR-TKIs such as gefitinib or erlotinib inhibit tumor cells by blocking the EGFR signaling via binding to ATP binding site of EGFR to increase survival in NSCLC patients [8, 24, 25]. Several clinical studies have confirmed that patients with *EGFR* mutation have benefited from EGFR-TKIs treatment [26–28]. There were two major EGFR-activating mutations including an in-frame deletion in exon 19 and an L858R substitution in exon 21, which account for about 90% of all clinically important mutations related to EGFR-TKIs sensitivity [29, 30]. Icotinib is a new type and the third world listed EGFR-TKIs to treat for NSCLC patients [8]. Several studies have revealed that *EGFR* mutant patients could get more benefit from icotinib treatment than *EGFR* wild type patients [18, 22, 31–33]. More evidences have the trend to imply that *EGFR* 19Del patients have better efficacy of icotinib than L858R patients [20, 34, 35]. However, there were some negative results against these views [7, 14, 17]. Furthermore, these studies were small sample size and retrospective study. Whether *EGFR* mutation status, especial *EGFR* 19Del and L858R, influence the efficacy of icotinib in NSCLC patients, it is still unclear now. Therefore, we carried out this meta-analysis to figure them out.

Icotinib is a new EGFR-TKI used in the treatment of NSCLC patients and it is only available in China. There were no randomized controlled trials on the efficacy of icotinib in NSCLC patients harboring different *EGFR* mutation status. Most of studies were cohort and retrospective studies enrolled small sample sizes. Therefore, there were some risks of bias such as high regarding adequate sequence generation and blinding.

The heterogeneities of studies are common in meta-analysis because of the different type of studies, methods or the selected case and controls. In our study, there was no heterogeneity when we pooling the ORs of ORRs in *EGFR* wild type and mutant patients, the ORs of DCRs in *EGFR* 19Del and L858R patients. Meta-analysis results showed significant between-study heterogeneity in pooling analysis about DCRs in *EGFR* wild type and mutant patients and the ORRs in *EGFR* 19Del and L858R patients. Therefore, Mantel-Haenszel random-effects model was used for analysis. In order to figure out the source of heterogeneity in pooling ORs of DCRs in *EGFR* wild type and mutant patients, we conducted subgroup analysis by NOS. After the subgroup analysis by NOS, there was no heterogeneity in NOS 6 and 5 groups, but there was heterogeneity in NOS 7 group (Figure 3C). In order to draw more cautious conclusion on *EGFR* status on icotinib efficacy in NSCLC patients, we also performed the sensitivity analysis. We found that excluded studies did not influence the overall effective size in DCRs analysis of *EGFR* mutant patients *vs*. *EGFR* wild type patients (Figure 7A). For heterogeneity of the pooling ORRs in *EGFR* 19Del and L858R patients, sensitivity analysis showed one publication (Pang Lin-Rong, 2014) [21] could influence the overall effective size in ORRs analysis of *EGFR* 19Del and *EGFR* L858R patients(Figure 7B). After we excluded this publication [21], there was no heterogeneity for pooling ORs analysis for ORRs in *EGFR* 19Del and *EGFR* L858R patients (I^2^ = 43.8%, *P* = 0.058) (Figure 3B). We also carried the publication bias analysis by funnel plots, Egger's test and Begg's test. The results showed there was publication bias for pooling ORs analysis for DCRs in the *EGFR* wild type patients and *EGFR* mutant patients. Negative results of studies may not be published. Other three groups of pooling ORs analysis showed no publication bias. The inconsistency of these studies may be due to source of patients, disease condition, publication qualities or other clinical issues. Further large sample multi-center studies and well-designed research are needed.

There were some limitations in this meta-analysis. First, the studies enrolled in our meta-analysis were limited. Slight publication bias may exist because the research having negative results may have not been published online. Second, most studies included in our meta-analysis were retrospective and cohort studies, which may have selection bias and blinding bias. The relative small subjects in studies also influence the meta-analysis results.

In conclusion, our meta-analysis suggests that compared with *EGFR* wild type patients, *EGFR* mutant patients have better ORRs and DCRs after icotinib treatment; *EGFR* 19Del patients have better ORRs after icotinib treatment than *EGFR* L858R patients. *EGFR* mutation status is a useful biomarker for evaluation of icotinib efficacy in NSCLC patients. Because of limited numbers of studies and small sample sizes included as well as the heterogeneity in our meta-analysis, more randomized and large-scale clinical trials are necessary to confirm our meta-analysis results.

## MATERIALS AND METHODS

### Study review and selection

We reviewed the databases including PubMed, EMBASE, Web of Science, Wanfang and CNKI to 14 Oct. 2016. The searching strategy was “icotinib” or “conmana” and “cancer or carcinoma or tumor”. Dr. Jian Qu and Dr. Ya-Nan Wang reviewed all relevant articles to identify potential eligible studies.

### Inclusion and exclusion criteria

The inclusion criteria were including as follows: clinical study on NSCLC patients harboring *EGFR* status (*EGFR* wild type is defined as no mutation for 19Del and L858R; *EGFR* mutation is defined as harboring *EGFR* 19Del or L858R mutation) using icotinib treatment; at least have the one clinical indicator (ORR and DCR). A study was excluded if it was not relevant to cancer and clinical patients, *EGFR* status, or had no clinical indicators; involved just in animals or cells; or was a review, or abstract having no data. Different opinions on selections were solved by all author's discussion.

### Data collection, quality assessment and assessment of risk of bias

Therapeutic efficacy was evaluated by response evaluation criteria in solid tumors (RECISTC) including complete response (CR), partial response (PR), stable disease (SD) and progressive disease (PD). Clinical outcome indicators include objective response rate (ORR) and disease control rate (DCR). The data on authors’ names, sex, smoking status. Two investigators (Dr. Qu Jian and Dr. Ya-Nan Wang) independently extracted *EGFR* mutation type, numbers and clinical outcomes. The Newcastle-Ottawa Scale (NOS) method was used to evaluate the quality of selected studies. According to the Cochrane Handbook version 5.1.0 [36], the risk of bias was assessed including method of random sequence generation (selection bias), allocation concealment (selection bias), blinding (performance bias and detection bias), incomplete outcome data (detection bias), and selective reporting (detection bias). We evaluated methodological quality as low, high, or unclear risk of bias.

### Statistical analysis

We used STATA version 12 (Stata Corp, College Station, TX, USA) to carry out the meta-analysis. Heterogeneity was assessed by the Cochrane's *Q*-statistic test and *I*^2^ test. We used a Mantel-Haenszel random effects model in the analysis if *P* < 0.05 and *I*^2^ > 50%, otherwise, a Mantel-Haenszel fixed effects model was chosen [37]. Count data calculate the relative risk (RR) or odds ratio (OR) and expressed as 95% confidence interval (CI). Publication bias was analyzed by funnel plot, Egger's test and Begg's test. Sensitivity analysis was performed to assess the influence of a single study on the overall effective size. Tests were two-sided and statistical significance was accepted at *P* < 0.05.
